# A rare case of soft tissue sarcoma in the supraclavicular region: A case report

**DOI:** 10.1002/ccr3.7211

**Published:** 2023-05-01

**Authors:** Ronald Kato, Solomon Kibudde, Yusuf Mwondha, Starlin Farah

**Affiliations:** ^1^ Department of Emergency Medicine Savannah Hospital Nairobi Kenya; ^2^ Department of solid tumors Uganda Cancer Institute Kampala Uganda; ^3^ Department of General Medicine Tendercare Hospital Nairobi Kenya

**Keywords:** angiogram, angiosarcoma, chondrosarcoma, head and neck, histology, osteosarcoma, predominance, soft tissue sarcoma, ultrasound‐guided biopsy

## Abstract

Sarcomas of the head and neck account for about 2% of all head and neck malignancies in adults. The median age at diagnosis is 50–54 years with a slight male predominance. The rarity of these sarcomas and lack of prospective trials make it difficult to reach valid conclusions. A 36‐year‐old woman was referred to our hospital because of an expanding non‐pulsatile mass in the right supraclavicular fossa with associated numbness and paresthesia of the right forearm. The patient reported that the mass had gradually enlarged for the past 5 months. She had no history of trauma or any chronic illnesses. A physical examination revealed a mass measuring approximately 7 cm × 5 cm above the right clavicle with no palpable thrill or bruit. Ultrasound‐guided biopsy was done and the histology report revealed soft tissue cells, fatty cells, and skeletal muscle cells; no tumor cells were identified. Magnetic resonance angiogram studies were made and revealed a highly vascularized supraclavicular mass. Under a multidisciplinary approach, the mass was resected. Head and neck sarcomas are relatively rare tumors and those of the head and neck account for about 2% of all head and neck malignancies and 4%–10% of all sarcomas in adults. The main histologic subtypes are rhabdomyosarcoma, osteosarcoma, chondrosarcoma, and angiosarcoma.

## 1 INTRODUCTION

This case report presents a rare case of soft tissue sarcoma in the supraclavicular region of a 36‐year‐old woman. The patient presented with an insidious onset with no predisposing factors.

Sarcomas of the head and neck account for about 2% of all head and neck malignancies and 4%–10% of all sarcomas in adults. The clinical and histologic makeup of any of these large series of head and neck sarcomas is highly dependent upon the nature of the reporting institution or the clinical practice of the reporting clinician. There are variations and limitations in reporting head and neck sarcomas depending on their demographics, histologic, and anatomical makeup. The median age at diagnosis for all head and neck sarcomas is 50–54 years. Most series report a slight male predominance. Any site within the head or neck can be the primary site for a sarcoma since connective tissue is ubiquitous.

Patients generally present with a palpable mass, especially in the neck, skin changes, or subsite‐specific symptoms, for example, hoarseness of voice, epistaxis, dysphagia, nasal obstruction, or cranial nerve deficits.^2^ However, our patient only presented with a palpable neck mass for 5 months.^5^

Performing a biopsy to obtain representative tissues for histological evaluation is an important component of the initial evaluation. However, the biopsy method can affect subsequent treatment and thus should be carefully planned in conjunction with the surgeon who will perform the definitive surgery.^2^ Positive surgical margins are an independent predictor of poor survival. Negative margins are difficult to achieve in the region of the head and neck because these sarcomas tend to grow in tight anatomical confines and in close proximity to vital neurovascular structures. Our patient had 90% of the tumor resected as 10% was adhering to the major neck vessels.^5^

The rarity of head and neck sarcomas and lack of prospective trials make it difficult to reach valid conclusions.

## 2 CASE REPORT

A 36‐year‐old woman was referred to our hospital because of an expanding non‐pulsatile mass in the right supraclavicular fossa. The patient reported that the mass had gradually enlarged for the past 5 months. She had no history of trauma or any chronic illness; however, she reported numbness and paresthesia of the right forearm. A physical examination revealed a mass measuring approximately 7 cm × 5 cm above the right clavicle with no palpable thrill or audible bruit.

Duplex ultrasound scanning confirmed a cyst‐like mass with pericystic fluid in the right supraclavicular fossa; Ultrasound‐guided biopsy was done and the histology report revealed soft tissue cells, fatty cells, and skeletal muscle cells; no tumor cells were identified.

We decided to start on three dexamethasone infusions to decompress the mass with 16 mg, each over 4 h.

The decompression took 72 h after which the patient registered mild improvement in the numbness and paresthesia of the right hand.

Surprisingly, the patient experienced rebound swelling and severe pain, and numbness in the right hand after 72 h. The mass did not respond to dexamethasone decompression infusion.

A final decision was made to excise and perform intraoperative studies on the mass.

Under general anesthesia, intraoperatively, we found a highly aggressive vascularized mass with tendency of bleeding.

**FIGURE 1:**
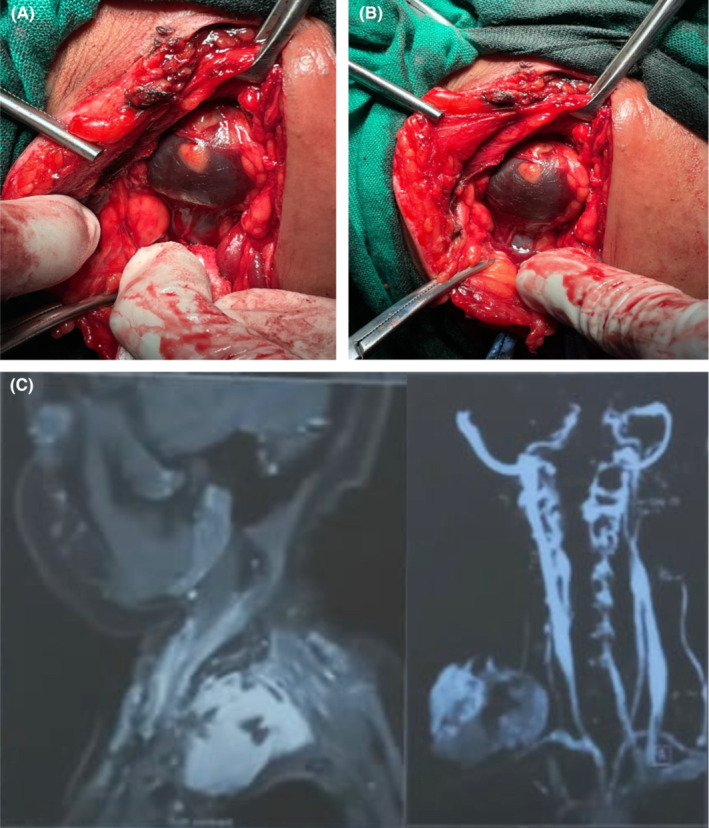
(A, B) Supraclavicular mass as seen intraoperatively. The mass extended well below the right clavicle; as such, the inferior surface of the mass could not be determined. **2.** ^5^ (C) Mass as seen on magnetic resonance angiography. Note that the mass has no relation to the right carotid artery.^4^

Intraoperative images were obtained (Figure 1A), and the mass could not be excised as it was extending posteromedially to the trapezius, inferomedially to the sternal border and medially to the trachea and great neck vessels. The base of the mass could not be ascertained.

**FIGURE 2:**
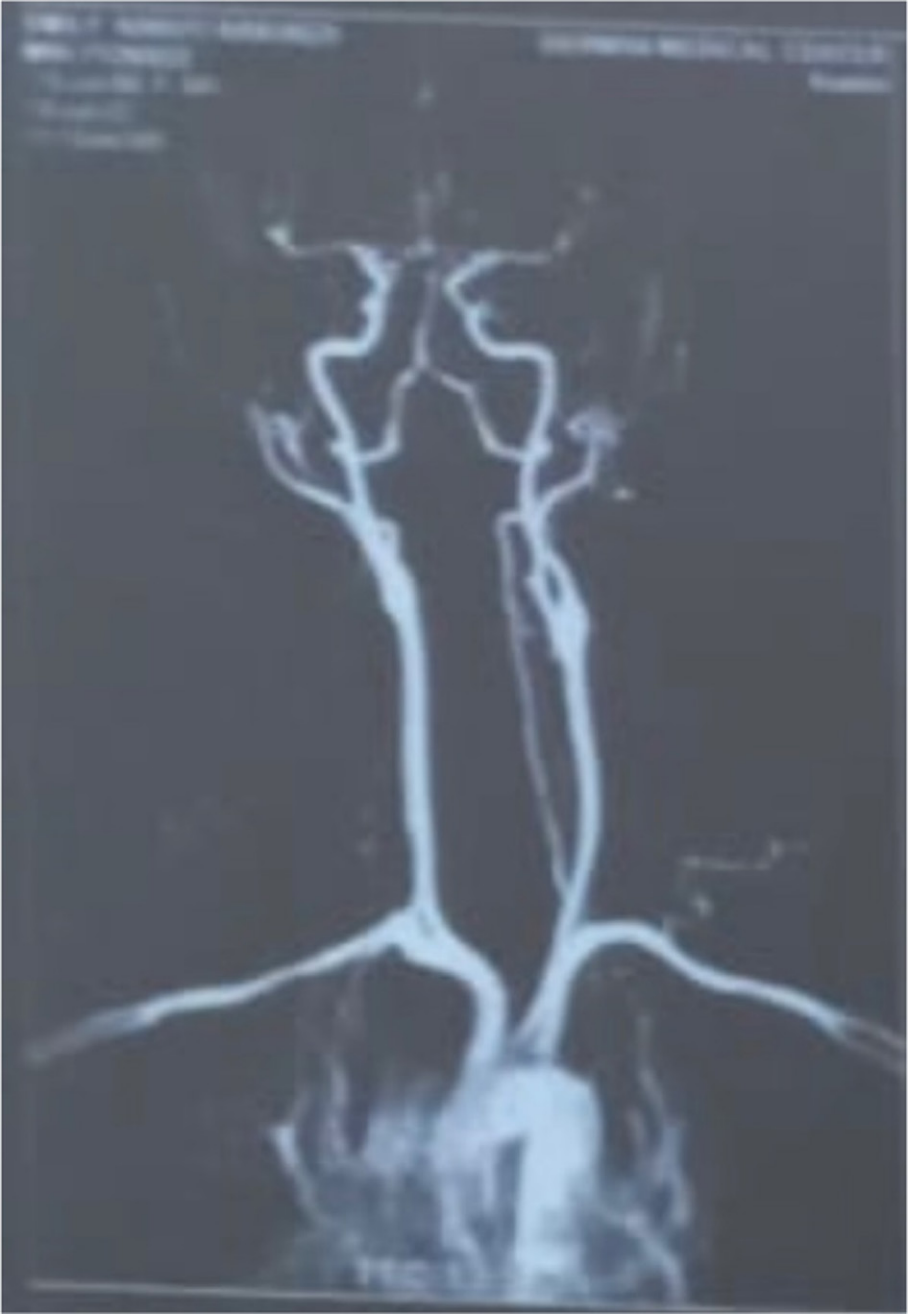
Magnetic resonance angiogram of the mass ascertaining no correlation of the mass to the right carotid artery.^5^

The decision to close and obtain magnetic resonance angiogram studies was made. Magnetic resonance imaging and angiography demonstrated a vascular mass compressing the brachial plexus, with no direct communication between the mass, the right subclavian artery and common carotids shown (Figures 1B and 2A).

A 3D reconstruction CT scan imaging was done to get proper approach and excision of the mass. The procedure was planned and incorporated a multidisciplinary team.

Under general anesthesia, the patient was placed in a beach chair position to obtain an adequate view of the mass. The right clavicle was removed by an orthopedic surgeon to expose the mass.

We dissected around the mass carefully so as not to damage the mass or surrounding vessels.

We worked from a distal approach on the subclavian artery segment toward the proximal exposure.

The feeding arteries were detected and divided with ligation which resulted in a decrease of pressure in the mass.

The mass had spread in most places and about 90% was resectable. The residual tissues could not be resected as they were far adhered to the major neck vessels.

Finally, the drainage veins were clamped after which the right clavicle was reconstructed with a plate by the orthopedic surgeon.

The histology report (H/8244/22) under H&E staining revealed the tumor is hypercellular and is composed of plump spindle cells with moderate pleomorphism. Mitotic count is 15–18/hpf and foci of necrosis are present. No residual normal tissue seen (Figure 2B).

**FIGURE 3:**
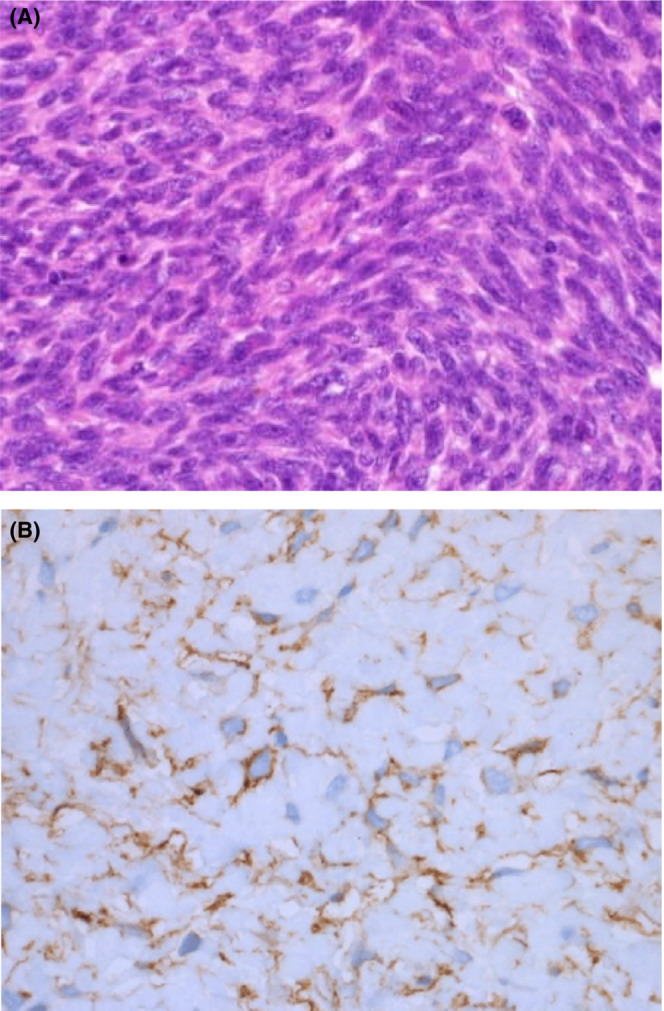
(A) Under H&E showing hypercellular plump spindle cells with pleomorphism and foci of necrosis.(B) CD34‐positive staining spindled‐to epithelioid cells with unusual glassy appearance.

The immunohistochemistry studies revealed CD34‐positive markers and composition of spindled to epithelioid cells with abundant eosinophilic cytoplasm with unusual glassy appearance (Figure 3A).

The patient was put in the intensive care unit for monitoring. The operation time was 4 h, and the estimated blood loss was 1000 mL.

## 3 CASE DISCUSSION

Head and neck sarcomas are relatively rare tumors and those of the head and neck account for about 2% of all head and neck malignancies and 4%–10% of all sarcomas in adults.

Sarcomas are malignant tumors arising from skeletal and extraskeletal connective tissues including the peripheral nervous system. Approximately 76% arise in soft tissue and the remainder in bone.

The main histologic subtypes are rhabdomyosarcoma in children, osteosarcoma, chondrosarcoma, and angiosarcoma.

The median age at diagnosis for all head and neck sarcomas is 50–54 years. Any site within the head or neck can be a primary site for a sarcoma, since connective tissue is ubiquitous.

There is no clearly defined etiology in most cases of soft tissue sarcoma, but several associated or predisposing factors have been identified. These include a genetic predisposition, gene mutations, radiation therapy (RT), chemotherapy, chemical carcinogens, chronic irritation, and lymphedema. In addition, an association between viral infection and sarcoma has been shown for HIV and human herpesvirus 8 in Kaposi's sarcoma, and for Epstein–Barr virus and smooth muscle tumors in immunocompromised patients.

The genetics of sarcomas segregate into two major types: those with specific genetic alterations and usually simple karyotypes, including fusion genes due to reciprocal translocations (e.g., PAX3‐FKHR in alveolar rhabdomyosarcomas) or specific point mutations (e.g., c‐kit mutations in gastrointestinal stromal tumors), and those with nonspecific genetic alterations and complex, unbalanced karyotypes, reflected by numerous genetic losses and gains (e.g., osteosarcoma, MFH, liposarcomas other than the myxoid type, angiosarcoma, leiomyosarcoma).

There is a high incidence of acquired (somatic) gene alterations in soft tissue and bone sarcomas. Somatic mutations in specific genes vary across histological subtypes of soft tissue sarcoma. Frequently mutated genes included the p53 tumor suppressor gene, NF1, and PI3KCA. DNA amplifications of chromosome 12q have been observed in 90% of patients with dedifferentiated liposarcomas.

Several other genes have been associated with soft tissue sarcomas.

1. The CD34 protein, the product of the CD34 gene (also called hematopoietic progenitor cell antigen CD34 gene), is expressed on the tumor cells of dermatofibrosarcoma protuberans (DFSP), but only on the vascular cells of malignant fibrohistiocytic lesions.
2. The expression of the HER2 (c‐erbB‐2) oncogene and the epidermal growth factor receptor is frequently and independently increased in bone and soft tissue sarcomas.

### 3.1 Differential diagnoses

The commonly used immunohistochemical markers for differential diagnosis of spindle cell neoplasms are smooth muscle actin, desmin, CD 34, S100 protein, endothelial markers, and melanocytic markers. The recognition of the spindle cell tumor‐like lesions (inflammatory myofibroblastic tumor, fascicular variant of pseudo‐angiomatous stromal hyperplasia) and tumors (myofibroblastoma, leiomyoma, schwannoma, spindle cell lipoma, and solitary fibrous tumor) is crucial to avoid confusion with morphologically similar but more aggressive tumors like myofibroblastic sarcoma and dermatofibrosarcoma protuberans.

Some very rare tumors like epitheloid hemangioendothelioma (EHE) is a rare vascular neoplasm of intermediate malignant potential, which commonly involves lungs, liver soft tissue, and bone. They usually present as an asymptomatic mass. Microscopically, they exhibit proliferation of epithelioid cells and spindle shaped endothelial cells. Epithelioid cells show cytoplasmic vacuoles with few cells containing RBCs.

**FIGURE 4:**
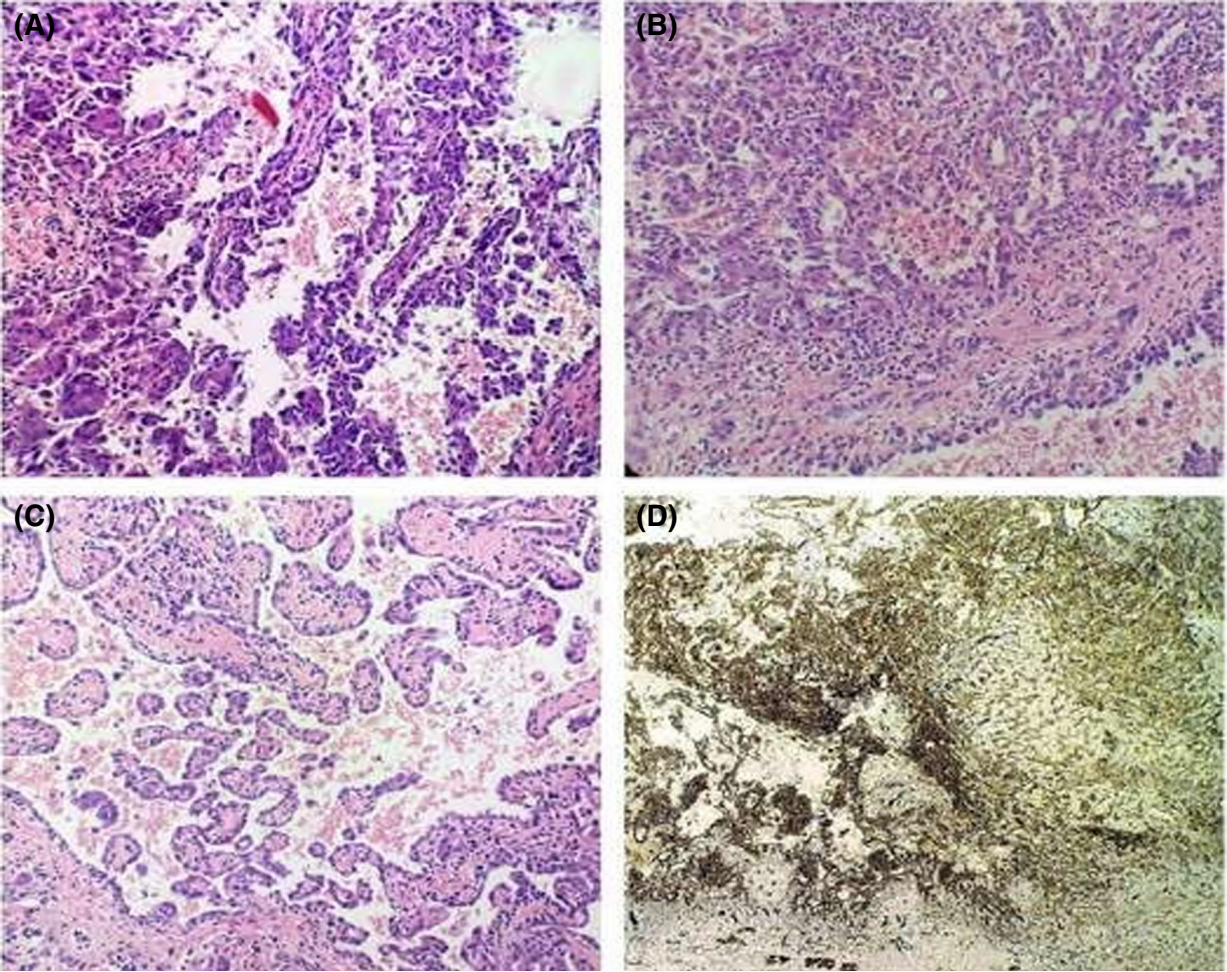
:(A) An uncapsulated lesion with numerous epithelioid cells and vascular areas (hematoxylin and eosin staining 20×). (B) Nests of neoplastic epithelioid cells with intracytoplasmic vacuolation (hematoxylin and eosin staining 20×). (c) Intracytoplasmic inclusion of epithelioid endothelial cells containing erythrocytes (hematoxylin and eosin staining 20×). (D) Positive immunohistochemical expression for CD 31 (40×).

A 34‐year‐old male presented to our institution with the chief complaint of swelling on the base of the tongue from 8 months. Surgical excision was done. An extensive workup of immunohistochemistry was done using different markers including CD 31, CD 34, Ki 67, Factor VIII, and BCL2. Correlation of histopathology and immunohistochemistry below confirmed the diagnosis of EHE (Figure 4).^3^, ^5^

### 3.2 Radiation therapy and chemotherapy

RT is recognized as a cause of sarcoma of bone and soft tissue. The most frequent histopathologic type of radiation‐induced soft tissue sarcoma arising in soft tissues is MFH. Some pathologists no longer use the term MFH and instead call these tumors undifferentiated pleomorphic sarcomas, pleomorphic fibrosarcoma, or pleomorphic myofibroblastic sarcomas. The frequency increases with the RT dose and with the postradiation observation period and decreases with age.

Sarcoma is primarily a complication of high‐dose therapy; it is rarely seen after low doses (<40 Gy). The actuarial frequency at 15–20 years is approximately 0.5% for RT of normal bone and soft tissue in adults treated with RT alone to full doses and without chemotherapy.

### 3.3 Prognosis

Secondary sarcomas have a poorer prognosis than do de novo sarcomas. As an example, in a comparison of 130 patients with radiation‐associated soft tissue sarcomas with a matched cohort of sporadic sarcomas, patients with a secondary sarcoma had significantly worse disease‐specific survival (HR 1.7, range 1.1–2.4). For MFH, 5‐year disease‐specific survival was 44% for patients with radiation‐associated sarcomas compared to 66% for patients with sporadic MFH.

## 4 CONCLUSION

Head and neck sarcomas are relatively rare tumors and those of the head and neck account for about 2% of all head and neck malignancies and 4%–10% of all sarcomas in adults. The median age at diagnosis for all head and neck sarcomas is 50–54 years. The main histologic subtypes are: rhabdomyosarcoma, osteosarcoma, chondrosarcoma, and angiosarcoma.

Several gene mutations, radiation therapy, chemotherapy, and viral infections contribute to sarcomagensis. The biopsy method can affect subsequent treatment and thus should be carefully planned in conjunction with the surgeon who will perform the definitive surgery.

Positive surgical margins are an independent predictor of poor survival. Negative margins are difficult to achieve in the region of the head and neck because these sarcomas tend to grow in tight anatomical confines and in close proximity to vital neurovascular structures.

The rarity of head and neck sarcomas and lack of prospective trials make it difficult to reach valid conclusions.

### PATIENT PERCEPTION ABOUT THIS CASE

The patient's perception is that the swelling was something that might be related to use of local herbs that she was using to treat cough. She also reported that the swelling was secondary to the wound she had on the right thumb that was treated by local herbal medicine.

## AUTHOR CONTRIBUTIONS

**Kato Ronald:** Conceptualization; investigation; methodology; resources; supervision; validation; writing – original draft; writing – review and editing. **Solomon Kibudde:** Formal analysis; supervision; validation. **Yusuf Mwondha:** Investigation; project administration. **Starlin Farah:** Formal analysis; resources; writing – review and editing.

## FUNDING INFORMATION

There was no funding or any financial assistance that was contributed toward this manuscript.

## CONFLICT OF INTEREST STATEMENT

There is no any conflict of interest amongst authors and all our institutions with regard to the publication of this report.

## CONSENT STATEMENT

Written informed consent was obtained from the patient to publish this report in accordance with the Journal's patient consent policy.

## Data Availability

The data that support the findings of this article are available in UptoDate at http://medup.ir/uptodate/contents/mobipreview.htm?31/18/32042?source=see_link Pathogenetic factors in soft tissue and bone sarcomas http://medup.ir/uptodate/contents/mobipreview.htm?33/50/34602 Head and neck sarcomas.
